# An AI based, open access screening tool for early diagnosis of Burkitt lymphoma

**DOI:** 10.3389/fmed.2024.1345611

**Published:** 2024-06-06

**Authors:** Nikil Nambiar, Vineeth Rajesh, Akshay Nair, Sunil Nambiar, Renjini Nair, Rajesh Uthamanthil, Teresa Lotodo, Shachi Mittal, Steven Kussick

**Affiliations:** ^1^Burkitt’s Lymphoma Fund for Africa, Seattle, WA, United States; ^2^Department of Comparative Medicine, University of Washington, Burkitt’s Lymphoma Fund for Africa, Seattle, WA, United States; ^3^Department Of Hematology and Blood Transfusion, Moi University/MTRH/AMPATH, Eldoret, Kenya; ^4^Department of Chemical Engineering, University of Washington, Seattle, WA, United States; ^5^Department of Laboratory Medicine and Pathology, University of Washington, Seattle, WA, United States; ^6^Department of Laboratory Medicine and Pathology, University of Washington, Burkitt’s Lymphoma Fund for Africa, Seattle, WA, United States

**Keywords:** Burkitt lymphoma, cancer, pediatric, pathology, artificial intelligence-AI

## Abstract

Burkitt Lymphoma (BL) is a highly treatable cancer. However, delayed diagnosis of BL contributes to high mortality in BL endemic regions of Africa. Lack of enough pathologists in the region is a major reason for delayed diagnosis. The work described in this paper is a proof-of-concept study to develop a targeted, open access AI tool for screening of histopathology slides in suspected BL cases. Slides were obtained from a total of 90 BL patients. 70 Tonsillectomy samples were used as controls. We fine-tuned 6 pre-trained models and evaluated the performance of all 6 models across different configurations. An ensemble-based consensus approach ensured a balanced and robust classification. The tool applies novel features to BL diagnosis including use of multiple image magnifications, thus enabling use of different magnifications of images based on the microscope/scanner available in remote clinics, composite scoring of multiple models and utilizing MIL with weak labeling and image augmentation, enabling use of relatively low sample size to achieve good performance on the inference set. The open access model allows free access to the AI tool from anywhere with an internet connection. The ultimate aim of this work is making pathology services accessible, efficient and timely in remote clinics in regions where BL is endemic. New generation of low-cost slide scanners/microscopes is expected to make slide images available immediately for the AI tool for screening and thus accelerate diagnosis by pathologists available locally or online.

## Introduction

Burkitt’s lymphoma (BL) is a relatively rare, aggressive form of non-Hodgkin’s lymphoma and the fastest growing human tumor with a doubling time reported to be about 24 h (1, 2). BL has a high prevalence in regions where malaria and Epstein–Barr virus (EBV) are endemic, particularly sub-Saharan Africa, where it represents up to 41% of all childhood cancers (3). The survival of pediatric BL patients is significantly lower in Africa (30–60% overall survival rate [OS]) compared to that of high-income countries (75–90% OS) (4, 5). Despite being highly curable (6), significantly higher mortality in BL in sub–Saharan Africa is caused by delayed or incorrect diagnosis (7) and limited efficacy of the available treatment regimens (5).

**Figure 1:**
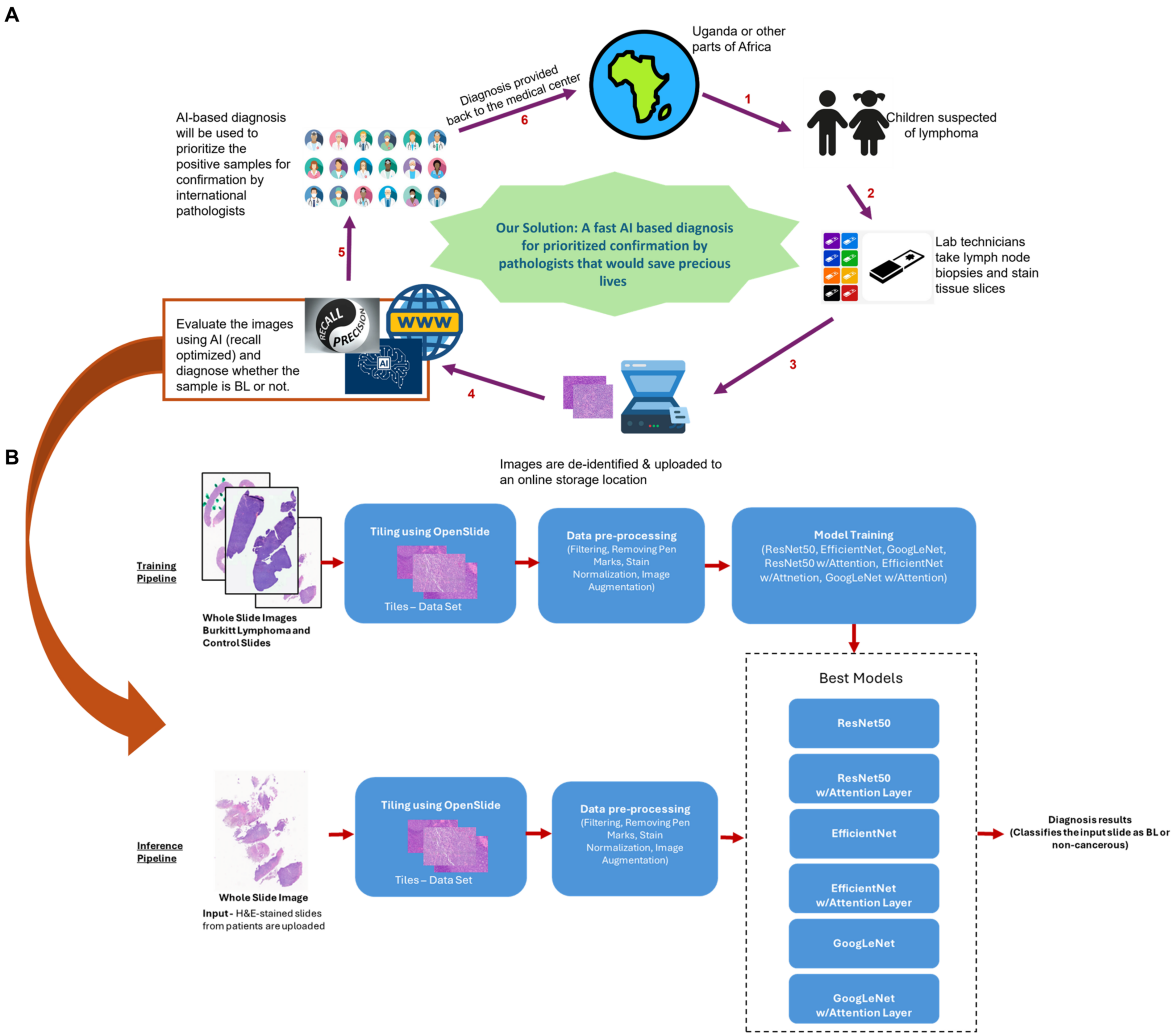
Proposed model for Al based preliminary diagnosis of BL. **(A)** For children suspected of BL, technicians would process tissue slices and upload de-identified whole slice images to an online secure storage location. This would trigger our Al pipeline resulting in an ensemble model based preliminary diagnosis being uploaded back to the storage. The files would then be accessed by a pathologist, who can use the Al based diagnoses to prioritize the samples for analysis and final diagnosis. **(B)** Curated H&E-stained whole slice images were preprocessed and used to train 6 models (ResNet50, EfficientNet and GoogLeNet, along with their attention enabled counterparts), generating an ensemble. For inferencing, the slice images uploaded to the storage would be similarly processed and scored by the ensemble model to generate a preliminary diagnosis. Icons in A were taken from www.fiaticon.com and www.istockphoto.com

Given how rapidly BL grows, early BL diagnosis is critical to reducing mortality, particularly in low-resource settings, where there is often limited ability to support late-stage, very ill patients compared to high-resource settings. Obstacles to histopathologic diagnosis of BL in Africa (1), include limitations in the resources to generate high-quality glass slides for microscopic review. This, combined with a severely limited number of pathologists available to provide timely diagnosis (7) creates significant delays and inefficiencies in diagnosis. To address this gap, we report on the development of an artificial intelligence (AI) tool focused on screening/preliminary diagnosis of BL using H&E slide images of different magnifications (Figure 1) (8–14). Leveraging the capabilities of contemporary deep learning techniques (15–17), we provide proof of concept for an AI model that would efficiently analyze histopathological images extracted from biopsy samples. The objective of the deep learning model we present here is to classify patient tissue sections as either BL or non-BL with high accuracy and sensitivity/recall (18–20). Our method is innovative in that it uses a significantly lower sample size (less than 160 patient samples) to train the model than is usually reported, but still captures the variance in the data and attains good performance on inference testing. Another novelty is that we use ensemble scoring, aggregating the classification results from six different models to provide a more accurate, robust and generalizable diagnosis.

The purpose of this AI model is to generate preliminary diagnoses and help international pathologists to prioritize cases for final diagnosis, i.e., to provide a screening tool that can accelerate timely and appropriate diagnosis of BL. The integration of our remotely accessible AI model into the healthcare infrastructure of low-resource settings should represent a significant stride forward in the fight against BL. While the model is accessible globally, the benefit should be particularly consequential to regions with scarce availability of specialized pathologists (5). By significantly reducing the diagnostic delays associated with traditional pathology and facilitating early detection and reliable diagnoses, this cutting-edge AI tool holds immense potential to help improve the management and outcomes of BL patients in sub-Saharan Africa.

## Materials and methods

### Data collection

As per University of Washington Institutional Review Board policy, this project did not need IRB approval as it did not meet federal definition of human subjects research. Whole Slide Images (WSI) of BL as well as benign control lymphoid tissues were obtained from Phenopath Laboratories (Seattle, United States) as well as Moi University, Kenya. The images were de-identified, ensuring the complete anonymity of the patients involved. These images encompassed a diverse array of cases, including lymph node samples from patients diagnosed with cancer from Africa, as well as tonsils samples from those without any cancer diagnosis. In total, our dataset consisted of 90 BL patients and 70 control (tonsillectomies or incisional biopsies of hypertrophic tonsils) patients. 7 BL slides were obtained from Moi University and the rest of the slides were obtained from Phenopath Laboratories. Slides were hematoxylin and eosin stained, scanned and digitized at 5X, 10X, 20X and 40X (i.e., 2 μm /pixel, 1 μm/pixel, 0.5 μm /pixel and 0.25 μm/pixel) magnifications. Motic@ Easyscan (Moi University) and an Aperio AT2 scanner (Phenopath) were used to scan the slides. For both the tonsil and BL samples, those with significant cautery or crush artifacts were excluded from the analysis.

### Data preprocessing

Our preprocessing pipeline starts by converting the raw WSI into smaller, manageable tiles using the OpenSlide library. These tiles were extracted at varying magnification levels, including 5x, 10x, 20x, and 40x, with specific tile sizes of 450×450 and 400×400 and a 20-pixel overlap to mimic the effect of a pathologist zooming in and out on the H&E slide to come up with a diagnosis.

A multi-step process was applied to filter and refine the dataset. Background tiles, with little to no tissue, were identified and outliers removed through a standard deviation-based filter applied to the RGB values of each tile. We detected and removed tiles with pen markings (21) made by pathologists by first converting RGB tiles into the HSV color space, where distinct color ranges for pen markings such as green, gray, and blue were established. Masks were created to isolate these specific colors, and a threshold was applied to filter out regions containing a significant amount of detected color, thus ensuring the automated removal of unwanted annotations.

Two common stains used are Hematoxylin, which dyes nuclei purple, and Eosin, which dyes cytoplasm and extracellular matrix pink. The regions with lower ratio of purple (Hematoxylin) to pink (Eosin) usually have scanty cellular material, extra cellular material (fibrosis) or artifacts such as folding or tearing. These areas might not provide reliable information for cancer diagnosis. Hence tiles with suboptimal Purple to Pink ratios were also filtered out using a scoring factor based on deviations in HSV hue values. Additionally, tiles with insufficient tissue presence were filtered using the Otsu thresholding method. While the previously mentioned standard deviation-based filtering focused on removing blank or near-blank tiles, the Otsu filtering focuses on separating foreground (tissue) from the background where the intensity distribution of the tissues is distinct from the background. Each tile was converted to grayscale, complemented, and then subjected to Otsu thresholding, with tiles not meeting the threshold being excluded.

Finally, we applied a normalization step (22, 23) to each tile, ensuring consistent color and intensity properties across all tiles and mitigating staining inconsistencies. These preprocessing steps ensured that the dataset was well-suited for our subsequent multi-instance learning (MIL) model analysis and make it robust to lab-lab stain variations.

### Computational setup

During our initial stages of pre-processing tasks and model training, we harnessed the capabilities of Google Colab Pro+, a cloud-based platform that provided convenient access to high-performance GPUs. Additionally, we relied on dedicated hardware equipped with a 13th Gen Intel Core i7-13700KF processor, boasting 16 cores and 24 logical processors, alongside an NVIDIA GeForce RTX 4080 GPU featuring 32GB of memory.

To harness the full potential of deep learning, we relied on the PyTorch framework for its flexibility and extensive libraries tailored for neural network development. Furthermore, Python served as our primary scripting language, indispensable for data preprocessing, model training, and the subsequent analysis of results. For processing Whole Slide Imaging (WSI), we utilized OpenSlide library, a valuable tool for reading WSI files and efficiently extracting patches or tiles.

### Model architecture

In the context of classifying WSI slides, the problem is often considered weakly supervised because obtaining precise annotations at the tile level is labor-intensive and often infeasible at scale. Generally, in classical weakly supervised (15, 24–26) analysis pipelines, all tiles within a slide inherit the same label, which can lead to suboptimal results when some tiles contain cancerous regions while others do not. In contrast, our approach leveraged the principles of Multiple-Instance Learning (MIL). In MIL, the training set consists of a bag of instances that are assigned a label even when the instances in that bag do not have a label associated with them. A bag is labeled positive if at least one instance in the bag is positive and is labeled negative otherwise. This enables a more nuanced classification, as some tiles within a slide may contain cancerous regions while others do not. MIL allows the model to learn from these heterogeneous instances within each slide.

To achieve an effective Multi-Instance Learning (MIL) framework (16, 17, 27, 28) for Whole Slide Image (WSI) classification, we thoroughly assessed various pretrained deep learning models. ResNet50, EfficientNet, and GoogLeNet (Inception-v1) emerged as the top models chosen for our MIL approach, considering performance and computational efficiency. To enhance their capabilities, we integrated attention layers (29) into these models, enabling them to focus on pertinent image regions, ultimately improving interpretability and classification performance.

ResNet50, renowned for its depth and residual blocks, gained the ability to dynamically emphasize relevant image regions with the addition of an attention layer. EfficientNet, known for its computational efficiency, leveraged attention to refine its fine-grained image analysis capabilities. GoogLeNet, celebrated for its inception modules, was augmented with an attention mechanism to enhance its interpretability and performance in capturing multi-scale features. These models, each equipped with attention layers, formed the core of our MIL framework to tackle the challenging task of WSI classification (Figure 1B).

Our MIL-based approach represents a novel and effective solution for WSI classification. Our approach does not rely on individual model predictions alone. Instead, we employ an ensemble strategy that combines the outputs of all six pretrained models, i.e., ResNet 50, EfficientNet and GoogLeNet, with and without attention as described above.

### Data splitting

To ensure balanced distribution and robust model training, we divided the dataset into training, testing, and validation subsets in a 6:2:2 ratio, employing a random selection process. Each bag in this context represented a single WSI, consisting of approximately 1,000 to 4,000 patches or tiles. These tiles, extracted during the preprocessing phase, encompassed various magnification levels, including 5x, 10x, 20x, and 40x, providing a comprehensive view of each WSI. For our overall training dataset, we compiled 63,323 tiles from the BL class and 117,375 tiles from the Control class, enabling our model to effectively learn the distinguishing features between BL and non-cancerous cases at different magnification levels.

### Training procedure

We conducted an extensive exploration of various training configurations to optimize the performance of each model. For every model, we conducted training runs with different batch sizes, ranging from 4 to 16, as well as varying sample sizes, encompassing 20 to 80 random samples selected from each bag during training. We maintained a consistent learning rate of 0.001 across all experiments. Prior to feeding the data into the training model, we applied preprocessing steps that included resizing each tile to a standardized dimension of 224×224 pixels and normalizing the images using mean values of [0.485, 0.456, 0.406] and standard deviations of [0.229, 0.224, 0.225].

We implemented an early stopping mechanism, wherein training was halted if the validation loss failed to decrease for five consecutive epochs (the number of times the model learns over the train data set). This strategy prevented overfitting and saved computational resources. Throughout the training phase, we continuously monitored model performance, saving the best-performing model for subsequent testing using CrossEntropyLoss as our loss function (30). The Adam optimizer was employed to efficiently update model parameters during training (30).

In our training loop for each of the six Multi-Instance Learning (MIL) models, we recorded both training and validation loss to gage the model’s progress. After experimenting with various training durations, we determined that training for 20 epochs struck a balance between model convergence and computational efficiency. This duration allowed our models to learn the intricate patterns within the data without overfitting or excessive training time, ultimately optimizing the trade-off between model performance and computational resources.

### Inference

The inference pipeline for our project is a multi-step process designed to classify Whole Slide Images (WSIs) efficiently and accurately into BL or non-cancerous categories. When a new WSI is uploaded for inference, the initial step involves the extraction of tiles at varying magnifications, including 5x, 10x, 20x, and 40x, which provides a comprehensive view of the slide.

Following tile extraction, a series of preprocessing steps, mirroring the training pipeline, are applied to enhance the quality of the extracted tiles and ensure consistent standardized color properties across the tiles. These steps involve filtering out blank tiles, tiles with pen markings, those with a lower purple to pink ratio and tiles with low tissue presence followed by normalization.

The heart of our inference pipeline involves the utilization of an ensemble model consisting of the six best machine learning models previously trained. Each of these models is employed to classify the image as either BL or non-cancerous based on the features extracted from the preprocessed tiles. To arrive at a final inference decision, the outputs of these individual models are considered collectively. This ensemble approach allows us to leverage the strengths of each model while mitigating potential biases or errors associated with a single model’s predictions. Ultimately, the final decision is determined based on a consensus among the ensemble of models, enhancing the robustness and reliability of our classification results (Figure 1).

### Evaluation metrics

In our evaluation of the models, we employed a range of performance metrics including Area Under the Curve (AUC), F1 score, Accuracy, and Recall (31). Accuracy score is the ratio of the number of correct predictions and the total number of predictions, while recall score indicates how many of the actual positive cases were predicted correctly with our model. The precision score explains how many of the correctly predicted cases turned out to be positive; F1 score is the harmonic mean of precision and recall and is maximum when precision is equal to recall. AUC scores provided us with a holistic view of the model’s ability to distinguish between different classes, specifically distinguishing between BL and Control (non-cancerous) cases. While these metrics offered a well-rounded perspective on the models’ performance, our emphasis was placed on optimizing recall. This choice was deliberate, by focusing on recall, we aimed to reduce the occurrence of erroneous categorizations where actual BL cases were incorrectly classified as Control, ultimately enhancing the model’s ability to detect true positive cases of BL, thus aligning with the overarching goal of this screening technique, i.e., reducing the number of misclassified true cancer instances.

## Results

### Model training and selection

**Figure 2:**
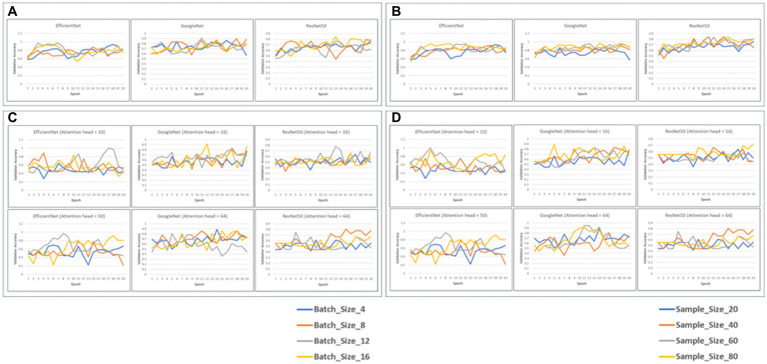
Validation accuracy of the 6 models with static batch size (4) or static sample size (20) **(A,B)**. The graphs depict the validation accuracies of EficientNet, Googl_eNet and ResNet60 for batch size4 **(A)** and sample size 20 **(C,D)**. Validation accuracies for the models with attention heads 10 and 50 for EfficientNet and 16 and 64 for GoogLaNet and RasNat60, with a batch size of 4 **(C)** and a sample size of (20) **(D)**.

We fine-tuned 6 pretrained models (EfficientNet, GoogLeNet and Resnet50, with and without attention layers) for a maximum of 20 epochs, validating performance on the validation data. We evaluated the training/validation losses and accuracies, and found that a batch size of 4 and sample size of 20 yielded best performance in terms of computational efficiency and validation loss/accuracy (Figure 2).

### Model performance

We evaluated the performance of all 6 models across different configurations, specifically focusing on static batch and sample sizes of 4 and 20, respectively (Tables 1–4). Our primary objective was to identify the configuration that yielded the best recall, while also considering a balanced trade-off between F1-Score and AUC, as our emphasis was on reducing the occurrence of erroneous categorizations, particularly false negatives. The performance of all models with and without attention across different configurations of batch, sample size, and attention head size is shown in Tables 1–4.

**Table 1:** Performance metrics of the 6 model variants; Accuracy, precision, recall, F1-score and AUC scores of the models with static batch size (4).

Model	Batch_Size	Sample_Size	Accuracy	Precision	Recall	F1-Score	AUC
ResNet50	4	20	0.67	0.75	0.68	0.71	0.66
ResNet50	4	40	0.67	0.86	0.55	0.67	0.7
ResNet50	4	60	0.53	0.73	0.36	0.48	0.57
ResNet50	4	80	0.5	0.67	0.36	0.47	0.54
EfficientNet	4	20	0.94	1.00	0.91	0.95	0.95
EfficientNet	4	40	0.72	0.69	1.00	0.81	0.64
EfficientNet	4	60	0.92	1.00	0.86	0.93	0.93
EfficientNet	4	80	1.00	1.00	1.00	1.00	1.00
GoogLeNet	4	20	0.94	0.92	1.00	0.96	0.93
GoogLeNet	4	40	0.81	0.94	0.73	0.82	0.83
GoogLeNet	4	60	0.83	0.81	0.95	0.88	0.80
GoogLeNet	4	80	0.94	0.92	1.00	0.96	0.93

The tables depict the metrics for EfficientNet, GoogLeNet and ResNet50 for batch size 4.

**Table 2:** Performance metrics of the 6 model variants; Accuracy, precision, recall, F1-score and AUC scores of the models with static sample size (20).

Model	Batch_Size	Sample_Size	Accuracy	Precision	Recall	F1-Score	AUC
ResNet50	4	20	0.67	0.75	0.68	0.71	0.66
ResNet50	8	20	0.89	0.95	0.86	0.9	0.9
ResNet50	12	20	0.75	1	0.59	0.74	0.8
ResNet50	16	20	0.78	0.94	0.68	0.79	0.81
EfficientNet	4	20	0.94	1.00	0.91	0.95	0.95
EfficientNet	8	20	0.94	0.92	1.00	0.96	0.93
EfficientNet	12	20	0.81	0.94	0.73	0.82	0.83
EfficientNet	16	20	0.89	0.85	1.00	0.92	0.86
GoogLeNet	4	20	0.94	0.92	1.00	0.96	0.93
GoogLeNet	8	20	0.72	1.00	0.55	0.71	0.77
GoogLeNet	12	20	0.83	0.94	0.77	0.85	0.85
GoogLeNet	16	20	0.72	1.00	0.55	0.71	0.77

The tables depict the metrics for EfficientNet, GoogLeNet and ResNet50 for sample size 20.

**Table 3:** Performance metrics for the models with attention heads 10 and 50 for EfficientNet and 16 and 64 for GoogLeNet and ResNet50, with a batch size of 4.

Model	Batch_Size	Sample_Size	Attention_Head_Size	Accuracy	Precision	Recall	F1-Score	AUC
ResNet50	4	20	16	0.69	0.82	0.64	0.72	0.71
ResNet51	4	40	16	0.56	0.71	0.45	0.56	0.58
ResNet52	4	60	16	0.56	0.80	0.36	0.50	0.61
ResNet53	4	80	16	0.56	0.71	0.45	0.56	0.58
ResNet54	4	20	64	0.58	0.63	0.77	0.69	0.53
ResNet55	4	40	64	0.72	0.73	0.86	0.79	0.68
ResNet56	4	60	64	0.61	0.90	0.41	0.56	0.67
ResNet57	4	80	64	0.81	1.00	0.68	0.81	0.84
EfficientNet	4	20	10	0.83	0.83	0.91	0.87	0.81
EfficientNet	4	40	10	0.92	0.88	1.00	0.94	0.89
EfficientNet	4	60	10	0.97	1.00	0.95	0.98	0.98
EfficientNet	4	80	10	0.58	0.89	0.36	0.52	0.65
EfficientNet	4	20	50	0.69	0.72	0.82	0.77	0.66
EfficientNet	4	40	50	0.69	1.00	0.50	0.67	0.75
EfficientNet	4	60	50	0.61	1.00	0.36	0.53	0.68
EfficientNet	4	80	50	0.97	1.00	0.95	0.98	0.98
GoogLeNet	4	20	16	0.56	0.65	0.59	0.62	0.55
GoogLeNet	4	40	16	0.61	0.83	0.45	0.59	0.66
GoogLeNet	4	60	16	0.81	0.86	0.82	0.84	0.80
GoogLeNet	4	80	16	0.94	0.95	0.95	0.95	0.94
GoogLeNet	4	20	64	0.81	0.94	0.73	0.82	0.83
GoogLeNet	4	40	64	0.81	0.89	0.77	0.83	0.81
GoogLeNet	4	60	64	0.75	0.74	0.91	0.82	0.70
GoogLeNet	4	80	64	0.78	1.00	0.64	0.78	0.82

**Table 4:** Performance metrics for the models with attention heads 10 and 50 for EfficientNet and 16 and 64 for GoogLeNet and ResNet50, with a sample size of 20.

Model	Batch_Size	Sample_Size	Attention_Head_Size	Accuracy	Precision	Recall	F1-Score	AUC
ResNet50	4	20	16	0.69	0.82	0.64	0.72	0.71
ResNet51	8	20	16	0.56	0.64	0.64	0.64	0.53
ResNet52	12	20	16	0.42	1.00	0.05	0.09	0.52
ResNet53	16	20	16	0.64	0.76	0.59	0.67	0.65
ResNet54	4	20	64	0.58	0.63	0.77	0.69	0.53
ResNet55	8	20	64	0.47	0.58	0.50	0.54	0.46
ResNet56	12	20	64	0.53	0.67	0.45	0.54	0.55
ResNet57	16	20	64	0.64	0.85	0.50	0.63	0.68
EfficientNet	4	20	10	0.83	0.83	0.91	0.87	0.81
EfficientNet	8	20	10	0.56	0.62	0.73	0.67	0.51
EfficientNet	12	20	10	0.94	1.00	0.91	0.95	0.95
EfficientNet	16	20	10	0.83	1.00	0.73	0.84	0.86
EfficientNet	4	20	50	0.69	0.72	0.82	0.77	0.66
EfficientNet	8	20	50	0.69	0.67	1.00	0.80	0.61
EfficientNet	12	20	50	0.97	0.96	1.00	0.98	0.96
EfficientNet	16	20	50	0.67	0.78	0.64	0.70	0.68
GoogLeNet	4	20	16	0.56	0.65	0.59	0.62	0.55
GoogLeNet	8	20	16	0.61	0.72	0.59	0.65	0.62
GoogLeNet	12	20	16	0.78	0.85	0.77	0.81	0.78
GoogLeNet	16	20	16	0.67	0.75	0.68	0.71	0.66
GoogLeNet	4	20	64	0.81	0.94	0.73	0.82	0.83
GoogLeNet	8	20	64	0.75	0.88	0.68	0.77	0.77
GoogLeNet	12	20	64	0.89	0.95	0.86	0.90	0.90
GoogLeNet	16	20	64	0.72	0.88	0.64	0.74	0.75

#### Models without the attention layers on the test data

##### ResNet50

The model demonstrated the best recall of 0.86 when using a batch size of 8 and a sample size of 20. This configuration also exhibited high precision (0.95), indicating a strong ability to classify true positives while minimizing false positives. The F1-Score and AUC of 0.90 reaffirmed the model’s capability to effectively differentiate between cancerous and non-cancerous cases. Therefore, this was chosen as the optimal model for the final classification.

##### EfficientNet

Again, the model demonstrated exceptional performance with a batch size of 8 and a sample size of 20, achieving a recall of 1.00. This configuration maintained strong precision (0.92), further underscoring its ability to accurately classify true positives. The resulting F1-Score of 0.96 and an AUC of 0.93 emphasized the well-rounded performance, combining precision and recall seamlessly. Thus, after evaluating the model’s performance across various configurations, the batch size of 8 and a sample size of 20 emerged as the most effective choice.

##### GoogLeNet

Among the configurations with static batch sizes, the model achieved the highest recall of 1.00 when employing a batch size of 4 and a sample size of 20. This configuration also maintained strong precision (0.92), with a resulting F1-Score of 0.96 and an AUC value of 0.93. In summary, the batch size of 4 and a sample size of 20 performed optimally on metrics and was chosen as the GoogLeNet version for inference (Tables 1, 2).

#### Models with attention layer on inference data

##### ResNet50 with attention layer

Overall, when evaluating the trade-off between Attention_Head_Size and model performance for static sample sizes, the configuration with an Attention_Head_Size of 64, a batch size of 4, and a sample size of 20 demonstrated the highest recall and a good balance between precision and F1-Score (Tables 3, 4).

##### EfficientNet with attention layer

In summary, when evaluating the trade-off between Attention_Head_Size and model performance for static sample sizes, the configuration with an Attention_Head_Size of 50, a batch size of 12, and a sample size of 20 emerged as the optimal choice (Tables 3, 4).

##### GoogLeNet with attention layer

The configuration with a Attention_Head_Size of 16, a batch size of 4, and a sample size of 80 was chosen to be the optimal performant model (Tables 3, 4).

### Composite model performance on inference data

**Figure 3:**
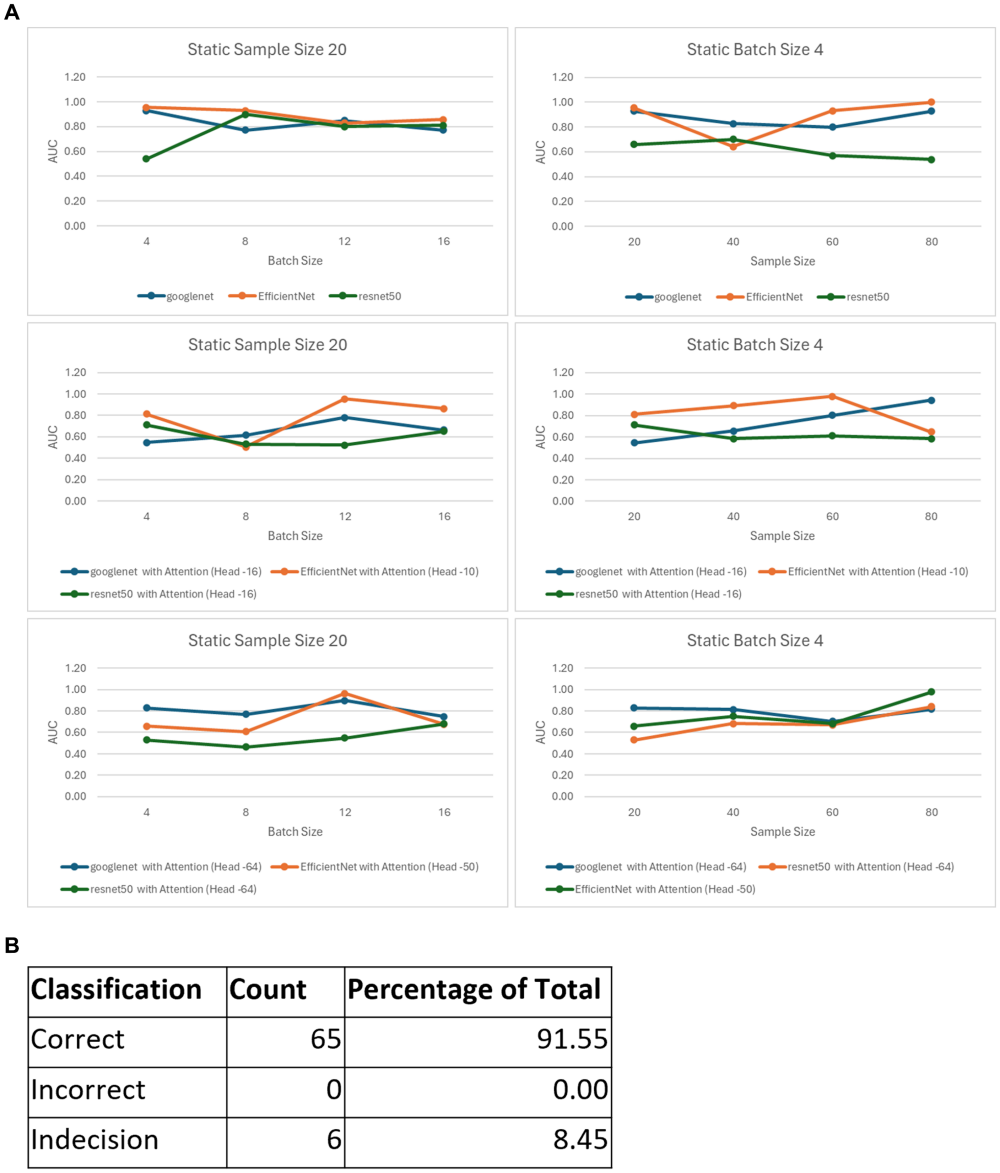
The ensemble model. **(A)** Best AUC scores for the variants of the 6 models, with those for static sample sizes shown in the first column and batch sizes in the second column. **(B)** Classification metrics for the ensemble model consisting of the 6 best performing variants.

After choosing the best configurations for all our six models, we formulated an ensemble model based on the best performers from the six pretrained model variants (with and without attention layers, Figure 3A). When the confidence score for the prediction was above 50% from the ensemble (i.e., when at least 4 models converged), the classification was counted as correct, and when it was below the threshold, it was counted as incorrect. When the confidence score was 50%, the classification was deemed not definite and termed ‘Indecision’. The performance was tested on this composite model for 35 samples, which classified 29 samples correctly (83%) and provided an indecision judgment for the other 6 samples (17%) due to low confidence (Figure 3B). The fact that none of the samples were classified incorrectly corroborates the high fidelity of our composite model.

## Discussion

The end-to-end flow of our model implementation begins when children are suspected of Burkitt lymphoma, prompting medical professionals to perform lymph node biopsies. These biopsies are formalin-fixed, paraffin-embedded, sectioned on to glass slides, and stained to enable the pathologist to distinguish BL from other diagnoses. As we envision our approach, Whole Slide Images (WSIs) would be acquired using state-of-the-art Whole Slide Scanners. Once acquired, and depending on the application, these images may go through a de-identification process to safeguard patient privacy before being uploaded to a secure online storage location. Prior to conducting any inference, the images would undergo an automated preprocessing pipeline to enhance the quality and relevance of the tiles within the images, mirroring the preprocessing steps employed during the training of our machine learning models. The inference pipeline involves the utilization of the six best-performing machine learning models that were previously trained for the task to give a composite score. Each of these models leverages the features extracted from the preprocessed tiles to classify the image as either Burkitt lymphoma (BL), non-cancerous or an indecision (Figure 1). The cases classified as BL or indecision by the AI model would be flagged for prioritized final diagnosis by relevant pathologists.

One of the innovative advances of our model is that by utilizing MIL with weak labeling and image augmentation, we were able to use a relatively low sample size to achieve an extremely good performance on the inference set. This means that the method efficiently captures the variance in the data without overfitting. We have used multiple magnifications for each sample, allowing the model to learn from multiple image magnifications, thus enabling inference and classification for a reasonable combination of magnifications (5x, 10x, 20x and/or 40x) a user is likely to provide.

Another novelty is the use of composite scoring with multiple models, essentially aggregating results from the models for this specific application. We experimented with adding attention layers to augment the original pretrained models, and the best models were chosen based on the assessment of performance with different configurations of the model hyperparameters (batch sizes) and image selection settings (sample sizes). This ensemble-based consensus approach ensures a balanced and robust classification, benefiting from the diverse strengths of each model. By achieving a consensus among these models, we enhance the reliability and accuracy of our final diagnosis. Our MIL-based approach, with its pretrained models enriched by attention mechanisms and empowered by ensemble learning, represents an innovative solution for precise and nuanced WSI classification, advancing BL diagnosis through automated WSI analysis.

The final AI based diagnosis outcome is communicated back to the medical center where the images were initially acquired (Figure 1). The aggregation of results from multiple good models provides more confidence in the preliminary diagnosis as depicted by the high fidelity of the ensemble for inference testing. The collective decisions of these models are integral since the preliminary diagnosis should greatly assist in allowing overworked pathologists to prioritize cases according to the likelihood of an urgent diagnosis such as BL. By helping ensure timely and accurate identification of BL, thereby facilitating prompt medical intervention and treatment, this AI approach is likely to be of particular benefit. Finally, and very importantly, once pilot studies in field settings are completed with further training the model, this AI approach can be applied to a variety of other urgent cancer diagnoses in resource-limited settings, allowing the development of a suite of diagnostic aids for patients of all ages.

There are many limitations and challenges to the model that need to be addressed before it is ready for deployment in the field. This proof of concept model was purposefully limited to a single comparison of reactive lymphoid tissue versus BL, to determine whether a relatively low-cost screen for the simple case of one type of benign process versus one type of malignancy might be feasible. Having convinced ourselves that this approach is feasible, we recognize that the next step in this process will require scanning additional BL cases and, more importantly, the following: (1) A broader range of non-tonsillar and non-malignant lymphoid proliferations (i.e., infectious/inflammatory processes); and (2) A variety of non-BL malignancies including other non-Hodgkin lymphomas, plus non-lymphoid small blue round cell tumors such as rhabdomyosarcoma, neuroblastoma, Wilm’s tumor, small cell carcinoma, and myeloid sarcoma. The model will need further training to learn potential variabilities in slide processing, staining as well as type of sample collection in various clinics/pathology labs in the region.

Future studies will involve collaborative projects with pathologists in BL endemic regions where the model is trained and tested with samples from clinical patients. These studies will also broaden the set of tumors to include a range of aggressive small blue round cell tumors of both hematopoietic and non-hematopoietic origin, and to broaden the set of benign/reactive tissues beyond tonsillar follicular lymphoid hyperplasia.

## Data availability statement

The original contributions presented in the study are included in the article/Supplementary material, further inquiries can be directed to the corresponding author.

## Ethics statement

As per University of Washington Institutional Review Board policy, this project did not need IRB approval as it did not meet federal definition of human subjects research. The studies were conducted in accordance with the local legislation and institutional requirements. The ethics committee/institutional review board waived the requirement of written informed consent for participation from the participants or the participants’ legal guardians/next of kin because no identifiable information (HIPPA) was obtained (only stained histology slides that has alternate IDs were used for the study).

## Author contributions

NN: Writing – review & editing, Writing – original draft, Visualization, Validation, Methodology, Investigation, Formal analysis, Data curation, Conceptualization. VR: Writing – review & editing, Writing – original draft, Visualization, Validation, Methodology, Investigation, Formal analysis, Data curation, Conceptualization. AN: Writing – review & editing, Writing – original draft, Visualization, Validation, Methodology, Investigation, Formal analysis, Data curation, Conceptualization. SN: Writing – review & editing, Writing – original draft, Visualization, Validation, Supervision, Methodology, Investigation, Data curation, Conceptualization. RU: Writing – review & editing, Writing – original draft, Validation, Supervision, Resources, Project administration, Methodology, Investigation, Conceptualization. RN: Writing – review & editing, Validation, Supervision, Resources, Investigation, Formal analysis, Conceptualization. TL: Writing – review & editing, Validation, Resources. SM: Writing – review & editing, Visualization, Validation, Supervision, Investigation, Data curation. SK: Writing – review & editing, Validation, Supervision, Resources, Methodology, Investigation, Formal analysis, Conceptualization.

## References

[1] López C Burkhardt B Chan JKC Leoncini L Mbulaiteye SM Ogwang MD. Burkitt lymphoma. Nat Rev Dis Primers. (2022) 8:78. doi: 10.1038/S41572-022-00404-3 36522349

[2] Ferry JA Wright JH. Lymphoma series: Variants of large-cell lymphoma Burkitt’s lymphoma: Clinicopathologic features and differential diagnosis. The oncologist (2006). 375–383. 10.1634/theoncologist.11-4-375 16614233

[3] Stefan C Bray F Ferlay J Liu B Parkin DM. Cancer of childhood in sub-Saharan Africa. Ecancermedicalscience. (2017) 11:1–95. doi: 10.3332/ecancer.2017.755, PMID: 28900468 PMC5574662

[4] Cairo MS Gerrard M Sposto R Auperin A Pinkerton CR Michon J. Results of a randomized international study of high-risk central nervous system B non-Hodgkin lymphoma and B acute lymphoblastic leukemia in children and adolescents. Blood. (2007) 109:2736–43. doi: 10.1182/BLOOD-2006-07-036665, PMID: 17138821 PMC1852225

[5] Buckle GC Collins JP Sumba PO Nakalema B Omenah D Stiffler K. Factors influencing time to diagnosis and initiation of treatment of endemic Burkitt lymphoma among children in Uganda and western Kenya: A cross-sectional survey. Infectious Agents and Cancer (2013). 10.1186/1750-9378-8-36 PMC3849966 24079452

[6] Eche-Ugwu IJ. Childhood Burkitt lymphoma in Nigeria. Clin J Oncol Nurs. (2023) 27:571–4. doi: 10.1188/23.CJON.571-574 37729460

[7] Gardie Y Wassie M Wodajo S Giza M Ayalew M Sewale Y. Delay in diagnosis and associated factors among children with cancer admitted at pediatric oncology ward, University of Gondar comprehensive specialized hospital, Ethiopia: a retrospective cross-sectional study. BMC Cancer. (2023) 23:469. doi: 10.1186/s12885-023-10873-8, PMID: 37217881 PMC10201796

[8] Niazi MKK Parwani AV Gurcan MN. Digital pathology and artificial intelligence. Lancet Oncol. (2019) 20:e253–61. doi: 10.1016/S1470-2045(19)30154-8, PMID: 31044723 PMC8711251

[9] Shmatko A Ghaffari Laleh N Gerstung M Kather JN. Artificial intelligence in histopathology: enhancing cancer research and clinical oncology. Nat Can. (2022) 3:1026–38. doi: 10.1038/s43018-022-00436-4, PMID: 36138135

[10] Tolkach Y Dohmgörgen T Toma M Kristiansen G. High-accuracy prostate cancer pathology using deep learning. Nat Mach Intell. (2020) 2:411–8. doi: 10.1038/s42256-020-0200-7

[11] Marini N Marchesin S Otálora S Wodzinski M Caputo A van Rijthoven M. Unleashing the potential of digital pathology data by training computer-aided diagnosis models without human annotations. NPJ Digit Med. (2022) 5:102. doi: 10.1038/s41746-022-00635-4, PMID: 35869179 PMC9307641

[12] Rashid R Chen YA Hoffer J Muhlich JL Lin JR Krueger R. Narrative online guides for the interpretation of digital-pathology images and tissue-atlas data. Nat Biomed Eng. (2022) 6:515–26. doi: 10.1038/s41551-021-00789-8, PMID: 34750536 PMC9079188

[13] Bera K Schalper KA Rimm DL Velcheti V Madabhushi A. Artificial intelligence in digital pathology — new tools for diagnosis and precision oncology. Nat Rev Clin Oncol. (2019) 16:703–15. doi: 10.1038/s41571-019-0252-y, PMID: 31399699 PMC6880861

[14] Madabhushi A Lee G. Image analysis and machine learning in digital pathology: challenges and opportunities. Med Image Anal. (2016) 33:170–5. doi: 10.1016/j.media.2016.06.037, PMID: 27423409 PMC5556681

[15] Dimitriou N Arandjelović O Caie PD. Deep learning for whole slide image analysis: an overview. Front Med (Lausanne). (2019) 6:2–4. doi: 10.3389/fmed.2019.00264, PMID: 31824952 PMC6882930

[16] Ilse M Tomczak JM Welling M. Attention-based deep multiple instance learning. Stockholm, Sweden, PMLR 80: Proceedings of the 35th International Conference on Machine Learning (2018).

[17] Chen CL Chen CC Yu WH Chen SH Chang YC Hsu TI. An annotation-free whole-slide training approach to pathological classification of lung cancer types using deep learning. Nat Commun. (2021) 12:1193. doi: 10.1038/s41467-021-21467-y, PMID: 33608558 PMC7896045

[18] Doeleman T Hondelink LM Vermeer MH van Dijk MR Schrader AMR. Artificial intelligence in digital pathology of cutaneous lymphomas: a review of the current state and future perspectives. Semin Cancer Biol. (2023) 94:81–8. doi: 10.1016/j.semcancer.2023.06.004, PMID: 37331571

[19] Chamba C Mawalla W. The future of lymphoma diagnosis, prognosis, and treatment monitoring in countries with limited access to pathology services. Semin Hematol. (2023) 60:215–9. doi: 10.1053/j.seminhematol.2023.07.004, PMID: 37596119

[20] Mremi A Achola C Mbwambo D Magorosa E Legason ID Vavoulis D. Diagnostic validation of a portable whole slide imaging scanner for lymphoma diagnosis in resource-constrained setting: a cross-sectional study. J Pathol Inform. (2023) 14:100188. doi: 10.1016/j.jpi.2023.100188, PMID: 36714453 PMC9874079

[21] Ali S Alham NK Verrill C Rittscher J. Ink removal from histopathology whole slide images by combining classification, detection and image generation models. Venice, Italy: IEEE International Symposium on Biomedical Imaging (ISBI) (2019).

[22] Macenko M Niethammer M Marron JS Borland D Woosley JT Guan X. A method for normalizing histology slides for quantitative analysis. Proceedings −*2009 IEEE international symposium on biomedical imaging: From Nano to macro, ISBI 2009*. (2009). p. 1107–1110.

[23] Pontalba JT Gwynne-Timothy T David E Jakate K Androutsos D Khademi A. Assessing the impact of color normalization in convolutional neural network-based nuclei segmentation frameworks. Front Bioeng Biotechnol. (2019) 7:2–6. doi: 10.3389/fbioe.2019.00300, PMID: 31737619 PMC6838039

[24] Campanella G Hanna MG Geneslaw L Miraflor A Werneck Krauss Silva V Busam KJ. Clinical-grade computational pathology using weakly supervised deep learning on whole slide images. Nat Med. (2019) 25:1301–9. doi: 10.1038/s41591-019-0508-1, PMID: 31308507 PMC7418463

[25] Wagner SJ Matek C Shetab Boushehri S Boxberg M Lamm L Sadafi A. Built to last? Reproducibility and reusability of deep learning algorithms in computational pathology. Mod Pathol. (2023) 37:5–9. doi: 10.1101/2022.05.15.22275108 37827448

[26] Ghaffari Laleh N Muti HS Loeffler CML Echle A Saldanha OL Mahmood F. Benchmarking weakly-supervised deep learning pipelines for whole slide classification in computational pathology. Med Image Anal. (2022) 79:102474. doi: 10.1016/j.media.2022.102474, PMID: 35588568

[27] Qu L Ma Y Luo X Wang M Song Z. Rethinking multiple instance learning for whole slide image classification: A good instance classifier is all you need. IEEE Transactions on Medical Imaging (2023). 10.1109/TMI.2024.3404549 38781068

[28] Li B Li Y Eliceiri KW. Dual-stream multiple instance learning network for whole slide image classification with self-supervised contrastive learning. 2021 IEEE/CVF Conference on Computer Vision and Pattern Recognition (CVPR). (2020). 10.1109/CVPR46437.2021.01409 PMC8765709 35047230

[29] Vaswani A Shazeer N Parmar N Uszkoreit J Jones L Gomez AN. Attention Is All You Need. Long Beach, CA, USA: 31st Conference on Neural Information Processing Systems (NIPS 2017) (2017).

[30] Ghosh J Gupta S. ADAM optimizer and CATEGORICAL CROSSENTROPY loss function-based CNN method for diagnosing colorectal Cancer. *2023 international conference on computational intelligence and sustainable engineering solutions (CISES)*. (2023). p. 470–474.

[31] Riehl K Neunteufel M Hemberg M. Hierarchical confusion matrix for classification performance evaluation. J R Stat Soc: Ser C: Appl Stat. (2023) 72:1394–412. doi: 10.1093/jrsssc/qlad057

